# Pericardial Metastasis From Recurrent Squamous Cell Carcinoma of Hypopharynx in a Living Patient

**DOI:** 10.7759/cureus.24216

**Published:** 2022-04-17

**Authors:** Drashti Antala, Khalid Mohamed, Leeseul Kim, Samir Bhatti, Pabitra Adhikari, Ira Oliff

**Affiliations:** 1 Internal Medicine, AMITA Health Saint Francis Hospital, Evanston, USA; 2 Radiology, AMITA Health Saint Francis Hospital, Evanston, USA; 3 Oncology, AMITA Health Saint Francis Hospital, Evanston, USA

**Keywords:** metastatic pyriform sinus carcinoma, pericardial metastasis, cardiac metastasis, squamous cell carcinoma, laryngopharyngeal cancer

## Abstract

Cardiac metastatic disease is a rare finding and is usually diagnosed incidentally postmortem; it has been commonly reported in patients with cancers of lung, esophagus, breast, and melanoma. We present a case of a 62-year-old male with a history of squamous cell carcinoma of the pyriform sinus who presented with shortness of breath for one day. He underwent tumor resection followed by chemotherapy and radiotherapy seven months before this presentation. Computed tomography (CT) of the chest revealed pericardial nodular soft tissue that was consistent with the diagnosis of metastatic carcinomatosis. Further imaging with a transthoracic echocardiogram (TTE) showed a likely metastatic pericardial mass. The patient had presented with shortness of breath three months prior to this admission and TTE had demonstrated pericardial effusion. However, pericardial fluid cytology was negative for malignancy, and the repeat TTE had revealed resolution of the pericardial effusion. On the current admission, CT of the neck demonstrated local recurrence of the tumor in the resection bed with scattered regional lymph nodes enlargement. Thus, we report a case of a recurrent laryngopharyngeal tumor with very rarely reported pericardial metastasis.

## Introduction

Squamous cell carcinoma of the pyriform sinus is the most common hypopharyngeal cancer and is usually diagnosed at an advanced stage due to a lack of obvious symptoms [1]. Given abundant lymphatic drainage of the hypopharynx, the incidence of lymph node metastasis of hypopharyngeal cancer is as high as 60% with a five-year overall survival of 30-35% [2,3]. 

Cardiac metastasis is a rare occurrence with a reported incidence of 1.23% among 12485 pan-cancer autopsies [4]. Hematologic or lymphatic spread is thought to be the most common mode of metastasis (67%) for right-sided chamber involvement and direct spread (64%) for pericardial involvement [5]. It is mainly diagnosed postmortem and is associated with an extremely poor prognosis with a median survival of 3.5 months without treatment. Treatment is mainly directed toward symptomatic management [6].

There have been very few reported cases of pharyngeal squamous cell carcinoma with cardiac metastasis. Here we report a rare case of cardiac metastasis of the recurrent pharyngeal squamous cell carcinoma found in a patient six months after completion of adjuvant chemoradiation following surgical resection of the primary tumor

## Case presentation

**Figure 1:**
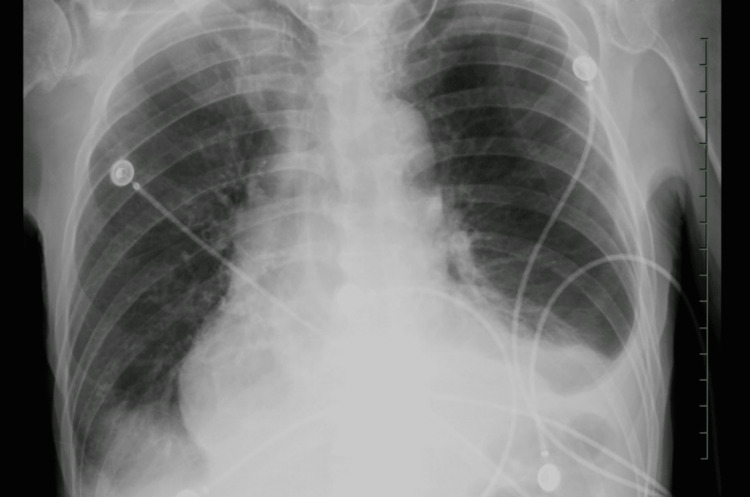
Chest x-Ray showing bibasilar hazy airspace opacities

A 62-year-old male with a history of pyriform sinus squamous cell carcinoma, and chronic obstructive pulmonary disease presented to the emergency department with acute shortness of breath for one day. He had undergone total laryngectomy with partial pharyngectomy and bilateral neck dissection with left supraclavicular flap reconstruction with stoma ten months prior, followed by adjuvant radiation and Cetuximab completed six months prior. He had a 100-pack-year smoking history. Vital signs were significant for hypotension with a blood pressure of 78/50 mm of Hg that improved after fluid resuscitation. Chest x-ray revealed bibasilar hazy airspace opacities (Figure 1).

**Figure 2:**
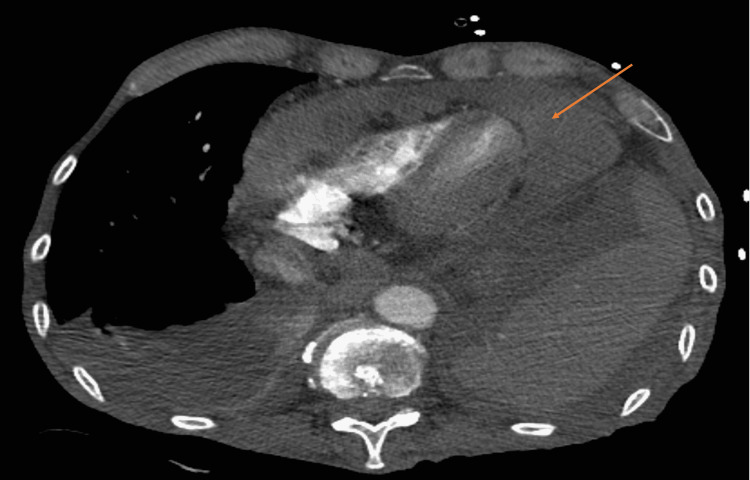
Diffuse nodular and thickened enhancing soft tissue replacement of the pericardium

Imaging with CT of the chest revealed marked nodular diffuse soft tissue replacement of the pericardium consistent with pericardial metastases with large interspersed pericardial effusion and numerous enlarged mediastinal lymph nodes (Figure 2).

**Figure 3:**
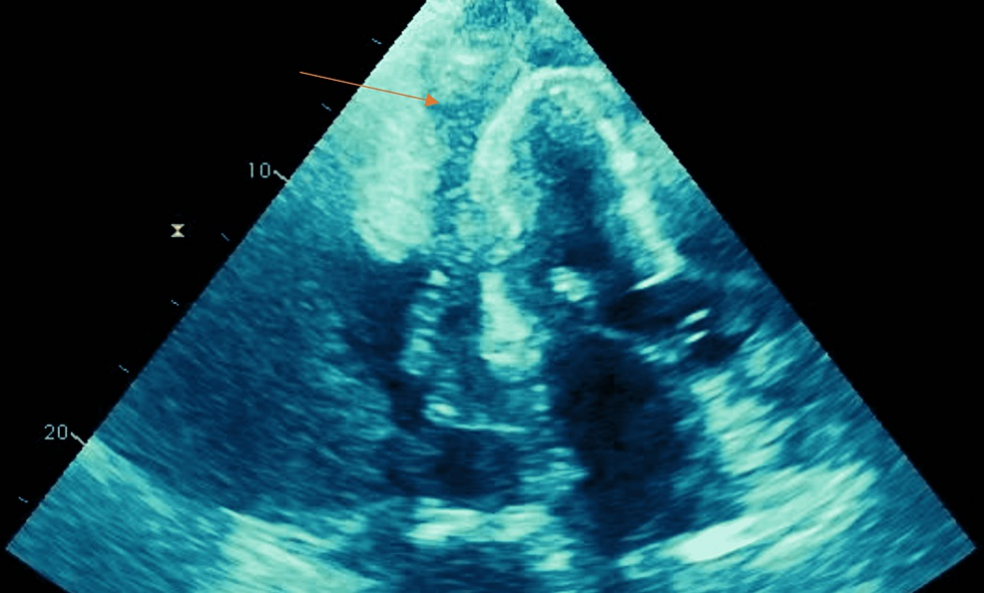
Transthoracic echocardiogram with echo dense mass in pericardial space

Transthoracic echocardiogram (TTE) showed small pericardial effusion with intermediate echo dense material noted in the pericardial space that was new compared to previous echocardiography findings (Figure 3).

Three months before this presentation, he had shortness of breath and TTE had shown moderate pericardial effusion without masses or tamponade physiology. Pericardial fluid analysis at that time was suggestive of exudate (protein in pericardial fluid sample 4.6 g/dl, serum protein 6.3 g/dl) but cytology did not reveal any malignant cells. Repeat TTE demonstrated resolution of the effusion following pericardiocentesis.

**Figure 4:**
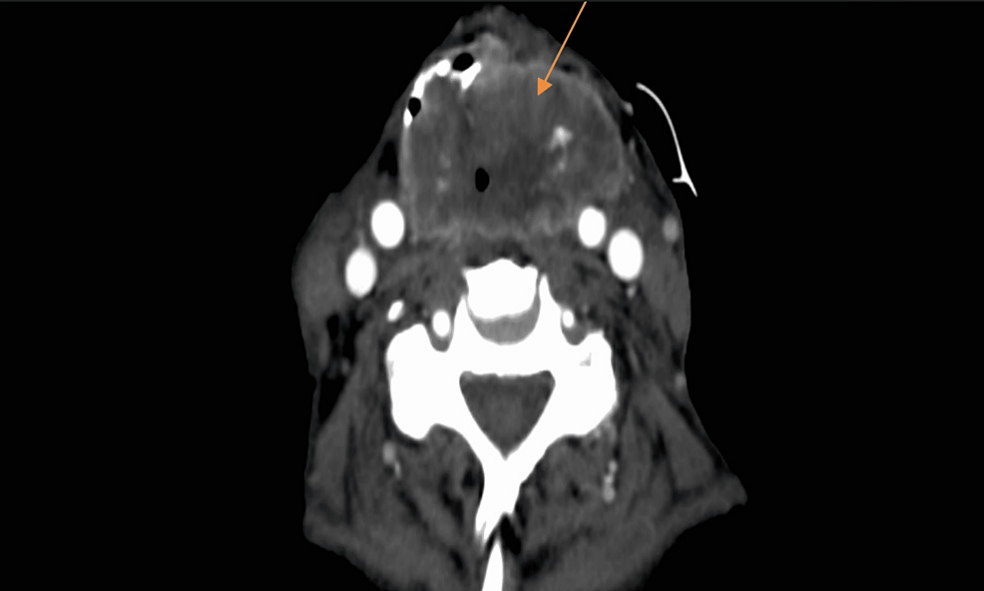
CT soft tissue neck with contrast with lobulated irregular enhancing mass in the expected location of pharynx and larynx

This admission, CT scan of neck demonstrated a large lobulated irregularly enhancing mass that measured 4.0 x 5.8 cm in anteroposterior and transverse dimension in the expected location of the pharynx and larynx that extended from the hypopharynx to the expected location of the lower larynx (mid C2-C5) (Figure 4).

**Figure 5:**
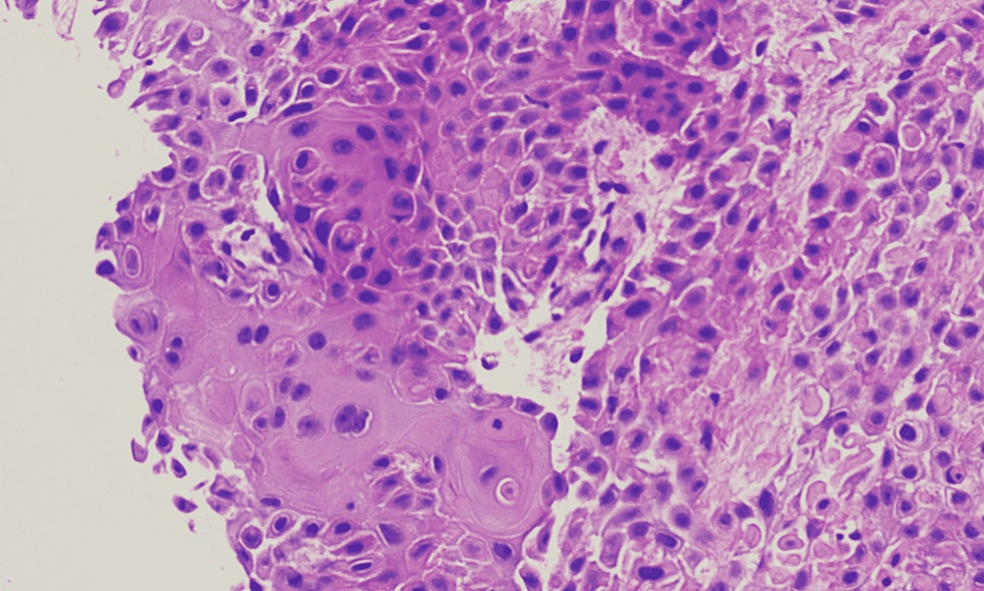
Core needle biopsy of neck mass consistent with squamous cell carcinoma on H and E stain

Neck mass biopsy was done which showed recurrent squamous cell carcinoma of the pharynx with invasion of the skeletal muscle (Figure 5).

He was planned to initiate immunotherapy, but no further treatment was given as the patient and his family opted against further aggressive therapy. 

## Discussion

Oropharyngeal squamous cell carcinoma (OPSCC) is a common type of head and neck cancer that accounts for over 13% of cases of all head and neck cancers globally [7]. Head and neck squamous cell cancers have locoregional growth predominantly with an incidence of distant metastasis relatively low compared to other malignancies such as stomach, lung, pancreas, etc. [8,9]. Distant metastasis to the heart is uncommon with literature mostly limited to case reports. Factors that have been linked to increased risk of distant metastasis include an advanced locoregional extension of the tumor, histological grade, advanced T- and N-classification, and tumor location at the hypopharynx or supraglottic region [9,10,11]. The risk of distant metastasis following definitive radiotherapy was found to be 14% at 5 years [12]. In a retrospective study, 5% of the patients with locoregionally controlled oral cavity, pharyngeal or laryngeal carcinoma died from the development of distant metastasis [10]. Detection of distant metastasis is associated with poor prognosis with a median survival of about 10 months [13]. 

Cardiac metastasis occurs infrequently, and the evidence is limited. Among all metastatic cases to the heart, pericardial involvement is the most common type of cardiac metastasis overall followed by epicardial and myocardial metastasis [14]. Tumors that have been most associated with metastasis to the pericardium include cancers of the lung, breast, melanoma, ovary, lymphoma, and mesothelioma [14,15]. Cardiac metastasis from head and neck squamous carcinoma is uncommon. Among cases of cardiac metastasis, oral cavity carcinoma had accounted for 5.3% of all cases in a study done by Bussani et al. [14]. Tumor cells invade the heart and pericardium by either retrograde lymphatic extension, hematogenous spread, transvenous, or direct local extension [15]. 

A high index of suspicion in the setting of a history of malignancy is needed for early detection of cardiac metastasis. Many of the patients might be asymptomatic or have some nonspecific symptoms which can end up being detected on autopsy [16,17]. When symptomatic, it can present as heart failure, arrhythmia, or cardiac tamponade [16]. Sometimes, massive pericardial effusion can be surrounded by tumors and cause persistent constriction, even after pericardiocentesis [14]. Echocardiography is the most common non-invasive test used to examine the heart and the pericardium. Additionally, CT and MRI can provide more details regarding disease involvement in the thorax, including the pleura, surrounding mediastinum, and vessels entering the heart [15]. MRI can assist in differentiating between the tumor and myocardium [15]. Given the rarity of the condition, the role of chemotherapy or radiation therapy is unclear. 

Our patient had presented three months before the current admission with shortness of breath secondary to pericardial effusion. He underwent pericardiocentesis and the fluid analysis did not reveal any malignant cells. The effusion was thought to be secondary to radiation. A repeat echocardiogram showed that the effusion had decreased in size. However, it remains possible that the effusion was due to malignancy with false-negative cytology. He was non-compliant with oncology follow-up for three months and later presented with shortness of breath. Contrast-enhanced cross-sectional imaging of the chest revealed diffuse nodular and thickened, enhancing soft tissue replacement of the pericardium. There was a significant mass effect upon the right ventricular outflow tract with obliteration of the typical pericardial fat planes, as well as bulky mediastinal lymphadenopathy. These imaging findings were new compared to imaging four months prior and pathognomonic for pericardial metastasis. Additionally, the patient underwent a contrast-enhanced CT of the neck which demonstrated a large, irregular, enhancing mass occupying the hypopharynx and larynx. 

Our patient underwent a biopsy of the neck mass rather than the pericardial mass as it was considered to be a safer procedure in the setting of his coexisting comorbidities. The biopsy showed recurrent squamous cell carcinoma. Given the pathologic diagnosis of recurrent SCC and the rapid development of the characteristic imaging findings on CT, there was sufficient evidence to reasonably conclude the presence of pericardial metastasis. This emphasizes the point that it might be beneficial to monitor echocardiogram in such patients with prior suspected malignant pericardial effusion, even in light of negative cytology but high risk of recurrence, as they might progress to developing widespread pericardial metastasis like our patient. The role of surveillance echocardiogram needs to be explored further and clinically correlated.

## Conclusions

Pericardial metastasis has been documented as one of the rare findings in patients with oropharyngeal cancer and it should be ruled out in such patients presenting with respiratory or cardiac symptoms. Development of pericardial mass may be preceded by the formation of effusion as in our case, and follow-up TTE might be beneficial for early detection and treatment with a very high index of suspicion. The overall prognosis and choice of treatment modality is unclear, which needs to be explored further. 
